# The Ferredoxin-Like Proteins HydN and YsaA Enhance Redox Dye-Linked Activity of the Formate Dehydrogenase H Component of the Formate Hydrogenlyase Complex

**DOI:** 10.3389/fmicb.2018.01238

**Published:** 2018-06-11

**Authors:** Constanze Pinske

**Affiliations:** Institute for Biology/Microbiology, Martin-Luther University Halle-Wittenberg, Halle, Germany

**Keywords:** [NiFe]-hydrogenase, formate hydrogenlyase, YsaA, HydN, formate dehydrogenase H, FDH-H, ferredoxin, FeS-cluster proteins

## Abstract

Formate dehydrogenase H (FDH-H) and [NiFe]-hydrogenase 3 (Hyd-3) form the catalytic components of the hydrogen-producing formate hydrogenlyase (FHL) complex, which disproportionates formate to H_2_ and CO_2_ during mixed acid fermentation in enterobacteria. FHL comprises minimally seven proteins and little is understood about how this complex is assembled. Early studies identified a ferredoxin-like protein, HydN, as being involved in FDH-H assembly into the FHL complex. In order to understand how FDH-H and its small subunit HycB, which is also a ferredoxin-like protein, attach to the FHL complex, the possible roles of HydN and its paralogue, YsaA, in FHL complex stability and assembly were investigated. Deletion of the *hycB* gene reduced redox dye-mediated FDH-H activity to approximately 10%, abolished FHL-dependent H_2_-production, and reduced Hyd-3 activity. These data are consistent with HycB being an essential electron transfer component of the FHL complex. The FDH-H activity of the *hydN* and the *ysaA* deletion strains was reduced to 59 and 57% of the parental, while the double deletion reduced activity of FDH-H to 28% and the triple deletion with *hycB* to 1%. Remarkably, and in contrast to the *hycB* deletion, the absence of HydN and YsaA was without significant effect on FHL-dependent H_2_-production or total Hyd-3 activity; FDH-H protein levels were also unaltered. This is the first description of a phenotype for the *E. coli ysaA* deletion strain and identifies it as a novel factor required for optimal redox dye-linked FDH-H activity. A *ysaA* deletion strain could be complemented for FDH-H activity by *hydN* and *ysaA*, but the *hydN* deletion strain could not be complemented. Introduction of these plasmids did not affect H_2_ production. Bacterial two-hybrid interactions showed that YsaA, HydN, and HycB interact with each other and with the FDH-H protein. Further novel anaerobic cross-interactions of 10 ferredoxin-like proteins in *E. coli* were also discovered and described. Together, these data indicate that FDH-H activity measured with the redox dye benzyl viologen is the sum of the FDH-H protein interacting with three independent small subunits and suggest that FDH-H can associate with different redox-protein complexes in the anaerobic cell to supply electrons from formate oxidation.

## Introduction

Ferredoxins are small proteins containing non-heme iron as iron-sulfur (FeS) clusters and they serve as electron carriers within the cell (Bruschi and Guerlesquin, 1988; Beinert, 1990). The group of ferredoxin proteins is ubiquitously distributed, very diverse and different classes can be distinguished based on the FeS cluster type (Bruschi and Guerlesquin, 1988; Beinert et al., 1997). They have typical cysteine motifs in common that co-ordinate the FeS clusters. In addition to the ferredoxin protein itself, Fdx, which is involved in FeS cluster synthesis, ferredoxin-like proteins often have functions as small or β-subunits of modular respiratory complexes like the *E. coli* proteins NarH of the nitrate reductase, HybA of the H_2_-oxidizing hydrogenase 2, FdoH and FdnH of the formate dehydrogenases O and N, respectively, NrfC of the periplasmic nitrite reductase, HycB and HycF of the H_2_-producing formate hydrogenlyase (FHL) complex and HyfA of the FHL-homologous complex, FHL-2. These proteins are predicted to have a similar core-fold with alpha-antiparallel beta sandwiches to hold the FeS-clusters (Pfam domain Fer4). In addition to the above, a number of other ferredoxin-like proteins are known in *E. coli*. Occasionally, these proteins are required for full activity of a particular respiratory enzyme, but are not essential components of the final enzyme. Among those are the NapF, G and H proteins, which are required for the full activity of the periplasmic nitrate reductase, and HydN, which is required for full formate dehydrogenase H activity (FDH-H) (Maier et al., 1996; Brondijk et al., 2002; Nilavongse et al., 2006). Other ferredoxin-like proteins like AegA and YsaA are not located within or close to an operon encoding an enzyme with predicted oxidoreductase activity and no phenotype has yet been described for mutants lacking these genes (Cavicchioli et al., 1996).

The FHL complex in *E. coli* is the main route of H_2_ production under fermentative growth conditions (Pinske and Sawers, 2016). The complex comprises a bis-molybdopterin guanine dinucleotide (Mo-bis-PGD)-dependent FDH-H that oxidizes formate to electrons and CO_2_. FDH-H is physically linked to the [NiFe]-hydrogenase protein HycE by 3 iron-sulfur (FeS) proteins. These are the FDH-H small subunit HycB, which has a ferredoxin-like fold and carries 4 FeS clusters, the ferredoxin-type protein HycF with 2 predicted FeS clusters, and the hydrogenase small subunit, HycG with a single FeS cluster. These five subunits are attached to the cytoplasmic side of the membrane by the HycC and HycD proteins. Notably, the *fdhF* gene, which encodes FDH-H, is located separately on the chromosome from the FHL-encoding *hyc*-operon, but it belongs to the same regulon (Sawers, 2005; Pinske and Sawers, 2016). Furthermore, it was established that FDH-H is the most loosely attached protein component of the FHL complex (McDowall et al., 2015). In addition to the Mo-bis-PGD cofactor, the FDH-H protein contains a selenocysteine and a FeS cluster, the latter of which requires insertion prior to attachment of FDH-H to the FHL complex (Boyington et al., 1997). A further function for FDH-H has been proposed as part of an alternative FHL-2 complex, encoded by the *hyf*-operon and expressed under different growth conditions in *E. coli* (Andrews et al., 1997; Trchounian et al., 2012). While FDH-H activity is associated with fermentative growth, the other formate dehydrogenases in *E. coli*, FDH-N and FDH-O, are associated with respiratory formate oxidation. FDH-N is active during nitrate respiration while FDH-O is active in the presence of both oxygen and nitrate (Sawers et al., 1991).

It has been suggested that HydN forms an additional pool of small subunits for FDH-H when it is not incorporated into the FHL complex (Sargent, 2016). Due to a high similarity with YfrA from *Proteus vulgaris*, the probable FeS subunit of fumarate reductase, it was further suggested that HydN could be involved in the electron transfer from formate to fumarate. However, a Δ*hydN* strain was investigated for its ability to transfer electrons from formate to fumarate, and proved to be unimpaired (Maier, 1997). Notably, the *hydN* gene is in an operon with the *hypF* gene (Maier et al., 1996) and HypF is one of the universal maturases that assemble the [NiFe]-cofactor. Due to its co-expression with *hypF*, the product of *hydN* was also suggested to be involved in H_2_-metabolism of the cell. Nevertheless, the co-occurrence of *hydN* and *hypF* is negligible in other organisms, while on the other hand *hydN* scores highly in its co-occurrence with *hycB* (0.778) and with *fdhF* (0.572) (Szklarczyk et al., 2015), suggesting a tight functional linkage.

HydN is predicted to harbor four [4Fe-4S] clusters and resembles a formate dehydrogenase small subunit similar to HycB of the FHL complex with which it shares 39% amino acid identity (52% similarity). Although both proteins share the same ferredoxin-like fold, suggesting a function in electron transfer, they cannot functionally replace one another (Maier, 1997). Interestingly, the *hydN* gene forms a transcriptional unit with the *fdhF* gene in the opportunistic pathogen *Serratia liquefaciens*. This FDH-H is similar to the *E. coli* protein, but it harbors a cysteine instead of the catalytic selenocysteine in its active site. A respective *E. coli* Cys variant is 20-fold less active than the SeCys protein (Pinske and Sawers, 2016). Nevertheless, fermentative gas production from glucose has been observed in *S. liquefaciens* (Brenner et al., 2006), but it was not yet identified whether this gas is CO_2_ or H_2_.

Due to the weak phenotype of the *hydN* deletion strain (Maier et al., 1996), a new homology search was conducted to identify other possible homologs, and revealed the ferredoxin-type protein YsaA (synonym YiaI) in *E. coli*, which shares 62% amino acid identity (72% similarity) with HydN and is therefore even more closely related to HydN than HycB. The *ysaA* gene co-occurrence with *fdhF* is 0.545 and thus in the same range as the *hydN*-*fdhF* co-occurrence (Szklarczyk et al., 2015). Therefore, the presence of YsaA might exhibit functional redundancy with HydN. Nothing is known about the *ysaA* gene and it is not located near any hydrogenase-associated genes. However, an interaction with NuoE, a protein of the closely to FHL related respiratory Complex I, which is required for the association of the diaphorase subunit, and with RclA (synonym YkgC) has been reported (Arifuzzaman et al., 2006). Furthermore, expression of the gene is 2.7 fold up-regulated under anoxic conditions (Kang et al., 2005). Therefore, we wanted to investigate the role of YsaA in H_2_ metabolism and to establish the interaction network of these ferredoxin-like proteins based on the hypothesis described above.

## Materials and methods

### Growth conditions and strain construction

Strains and plasmids used in this study are listed in Table 1. Strains were routinely grown in liquid LB medium in a shaking incubator or on LB agar plates. For determination of enzyme activities and bacterial two hybrid interactions, the bacteria were grown in buffered TGYEP medium supplemented with glucose: 1% (w/v) trypton, 0.5% (w/v) yeast extract, 0.8% (w/v) glucose, 0.1 M potassium buffer, pH 6.5; according to Begg et al. (1977). The medium was supplemented with 100 μg ml^−1^ ampicillin to ensure plasmid maintenance. Furthermore, when required, chloramphenicol was used at 12.5 μg ml^−1^ and kanamycin at 50 μg ml^−1^. The strain DHD-N was used as Δ*hydN* mutant and was previously described (Maier et al., 1996). The *ysaA* deletion was constructed in BW25113 transformed with the lambda red recombinase plasmid pKD46 according to Datsenko and Wanner (2000) by using the oligonucleotides ysaA_5'KO 5′-CTCTGGCACTCTGCTGTTTTAGTGCAAAGGAGTGATCATG CCATGGTCCATATGAATATCCTCC-3′ and ysaA_3′KO 5′-CGCACTGTTCCGGCGTTGAGAAACGCCGGAAAACGTTTCA GCGATTGTGTAGGCTGGAGCT-3′ to amplify the *cat* gene from pKD3. The mutation was subsequently moved to MC4100, DHD-N and JW2694 (Δ*hycB*) by P1_vir_ phage transduction (Miller, 1972). The resistance cassette was eliminated using the pCP20 plasmid as described (Cherepanov and Wackernagel, 1995). The deletion of *aegA* was introduced by the method of Hamilton et al. (1989) by cloning the upstream region as KpnI/BamHI fragment with the oligonucleotides DaegA1_KpnI 5′-GCGGGTACCGCCTGATACCACGGCAAATC-3′ and DaegA2_BamHI 5′-GCGGGATCC CATAATAAAACGATTCATAAC-3′ and the downstream region as BamHI/HindIII fragment with the oligonucleotides DaegA3_BamHI 5′-GCGGGATCCCAGTCAAATCTCACTGATAG-3′ and DaegA4_HindIII 5′-GCGAAGCTTCGCCGGTTTTGATCATCTCC-3′ into pMAK705 and recombining with the desired target strain as described. Introduction of the Δ*hycB* deletion in DHD-N backgrounds or *vice versa* has been done according to Datsenko and Wanner (2000) by introducing the lambda red recombinase on the pKD46 plasmid, growing competent cells and inducing them with 10 mM L-arabinose before electroporation of a PCR fragment containing a kanamycin resistance cassette and the upstream and downstream regions of the gene. These PCR fragments were obtained after amplification of the corresponding regions from strain JW2694 (Δ*hycB*) or JW2683 (Δ*hydN*). All clones were verified using colony PCR.

**Table 1:** Strains and plasmids.

**Strain**	**Genotype**	**Reference/source**
MC4100	F^−^*araD139* Δ(*argF-lac*)*U169 ptsF25 deoC1 relA1 flbB150^−^ rspL150*^−^	Casadaban, 1976
BW25113	F^−^Δ (*araD-araB*)*567* Δ *lacZ4787*(::*rrnB-3*) λ^−^*rph-1* Δ (*rhaD-rhaB*)*568 hsdR514*	Baba et al., 2006
DHD-N	As MC4100, but Δ*hydN*	Maier et al., 1996
JW2683	As BW25113, but Δ*hydN*::*kan*	Baba et al., 2006
JW2694	As BW25113, but Δ*hycB*::*kan*	Baba et al., 2006
CPH090	As BW25113, but Δ*ysaA*	This work
CPH010	As MC4100, but Δ*ysaA*	This work
CPH011	As MC4100, but Δ*hydN* Δ*ysaA*	This work
CPH012	As MC4100, but Δ*aegA*	This work
CPH013	As DHD-N (Δ*hydN*), but Δ*aegA*	This work
CPH014	As CPH010 (Δ*ysaA*), but Δ*aegA*	This work
CPH015	As JW2694 (Δ*hycB*), but Δ*ysaA*	This work
CPH016	As CPH015 (Δ*hycB* Δ*ysaA*), but Δ*aegA*	This work
CPH017	As JW2694 (Δ*hycB*), but Δ*aegA*	This work
CPH018	As DHD-N (Δ*hydN*), but Δ*hycB*	This work
CPH019	As CPH015 (Δ*hycB* Δ*ysaA*), but Δ*hydN*	This work
CPH020	As MC4100, but Δ*hyaB* Δ*hybC* Δ*hycAI* Δ*fdhF*	This work
RM102	As MC4100, but Δ (*srl-recA*)*306*::Tn*10 fnr zci*::Tn*10*	Birkmann et al., 1987
BTH101	F′, *cya*-99, *araD*139, *galE*15, *galK*16, *rpsL*1(Str^R^), *hsdR*2, *mcrA*1, *mcrB*1	Karimova et al., 1998
**PLASMIDS**^a^		
pCP20	*FLP*^+^, λcl857^+^, λ*p*_R_ Rep^ts^, Amp^R^, Cm^R^	Cherepanov and Wackernagel, 1995
pKD46	Contains λ Red genes γ, β and *exo*; Amp^R^	Datsenko and Wanner, 2000
pJET1.2	Commercially available cloning vector; Amp^R^	Thermo Fisher Scientific
phydN	pBluescriptSK(+) containing *hydN* in BamHI and EcoRI site; Amp^R^	This work
pysaA	pJET1.2 containing *ysaA* in MCS; Amp^R^	This work
pT25	Bacterial two hybrid plasmid expressing the T25 fragment and a MCS at the 3′ end of T25; Cm^R^	Karimova et al., 1998
pT25-zip	pT25, Leucine zipper fused to T25 fragment (1–224 amino acids of CyaA)	Karimova et al., 1998
pT18	Bacterial two hybrid plasmid expressing the T18 fragment and a MCS at the 5′ end of T18; Amp^R^	Karimova et al., 1998
pT18-zip	pT18, Leucine zipper fused to T18 fragment (225–399 amino acids of CyaA)	Karimova et al., 1998

a *Further plasmids from the bacterial two hybrid system that were constructed here can be found in Table S2*.

### Cloning of *ysaA* and *hydN*

Cloning of *ysaA* gene in the pJET1.2 cloning vector (commercially available from Thermo Fisher Scientific) was done by using the T18-ysaAFW_HindIII 5′-GCGAAGCTTGATGAACCGGTTTATTATTGCG-3′ and T18C/T25-ysaARW_KpnI 5′-GCGGGTACCTTATCAAACAGGCTGCTGCCGTAGC-3′ oligonucleotides for amplification from chromosomal DNA of MC4100 by the Q5-DNA polymerase (NEB) according to the manufacturer's instructions. The *hydN* gene was cloned into pBluescriptSK(+) by using the oligonucleotides HydN_FW_BamHI 5′-CGCGGATCCATGAACCGTTTCATCATTGC-3′ and HydN_RW_EcoRI 5′-CGCGAATTCTTAGAACATCAGCGCCGT-3′, digestion of both vector and PCR product with BamHI and EcoRI and subsequent ligation. All cloning products were verified by sequencing.

### Bacterial two hybrid interactions

The bacterial two-hybrid system (Karimova et al., 1998) was used to clone constructs by amplifying the respective gene fragment from chromosomal DNA of MC4100 and digesting both the pUT18 or the pT25 target vectors and the insert with the same restriction enzymes before ligation. The bacterial two hybrid vectors were constructed to yield functional in-frame fusion proteins and all inserts were verified by sequencing. The oligonucleotides used are listed in Table S1, where the restriction sites are given as part of the oligonucleotide name. Generally, the entire orf was amplified except for the start codons (T25 fusions) or stop codons (T18 fusions) and all newly generated constructs are listed in Table S2. Plasmids of pT25 origin were transformed together with a pUT18 plasmid into BTH101 and grown as anaerobic culture in TGYEP, pH 6.5 at 30°C for 16h containing both ampicillin and chloramphenicol as antibiotics. Determination and calculation of β-galactosidase activity was done according to Miller (1972). Each experiment was performed three times independently, and the activity for each sample was determined in triplicate. Alternatively, a volume of 5 μl of the culture was spotted on McConkey plates containing 0.5% (w/v) maltose, the antibiotics ampicillin and chloramphenicol. The plates were then aerobically incubated at 30°C for 16 h.

### Enzymatic assays

For determination of protein activities, the cells were grown in TGYEP medium, pH 6.5 as standing liquid cultures in 50 ml reaction tubes at 30°C for 16 h. For induction of NAR and FDH-N enzyme synthesis, sodium nitrate was added to the anaerobic cultures to a final concentration of 1% (w/v). The cells were harvested by centrifugation, sonicated (20 W, 0.5 s pulses, 3 min) and the extracts used directly. Activities of the soluble formate dehydrogenase and total hydrogenase were measured as formate-dependent benzyl viologen (BV) reduction (FDH-H) and H_2_-dependent BV reduction (hydrogenase), respectively at 600 nm as described in Pinske et al. (2011a). Detection of NAR and FDH-N activities by in-gel activity staining was done as described (Pinske and Sawers, 2012). Immunoblotting against FDH-H (1:3000) polypeptides was done after denaturing gel electrophoresis as described (Pinske et al., 2011a).

### GC analysis of gases in culture headspace

For assessing the activity of the intact FHL complex, the H_2_ content of the gas headspace was quantified. The H_2_ content of the 10 ml gas phase of an overnight culture was measured by sampling 200 μl in a GC-2010. The system was equipped with a packed column (Shin Carbon Micropacked column ST80/100). The carrier gas was N_2_ with a flow rate of 13.9 ml min^−1^, the injector was kept at 140°C, the column at 110°C and the TCD detector at 150°C and 40 mA. Quantification was done with a calibration curve of known amounts of H_2_.

### Bioinformatics

The unrooted tree was created based on an alignment conducted within Uniprot (Clustal Omega) and visualized with iTOL (Sievers and Higgins, 2013; Letunic and Bork, 2016).

## Results and discussion

### HydN has paralogues in *E. coli*

**Figure 1:**
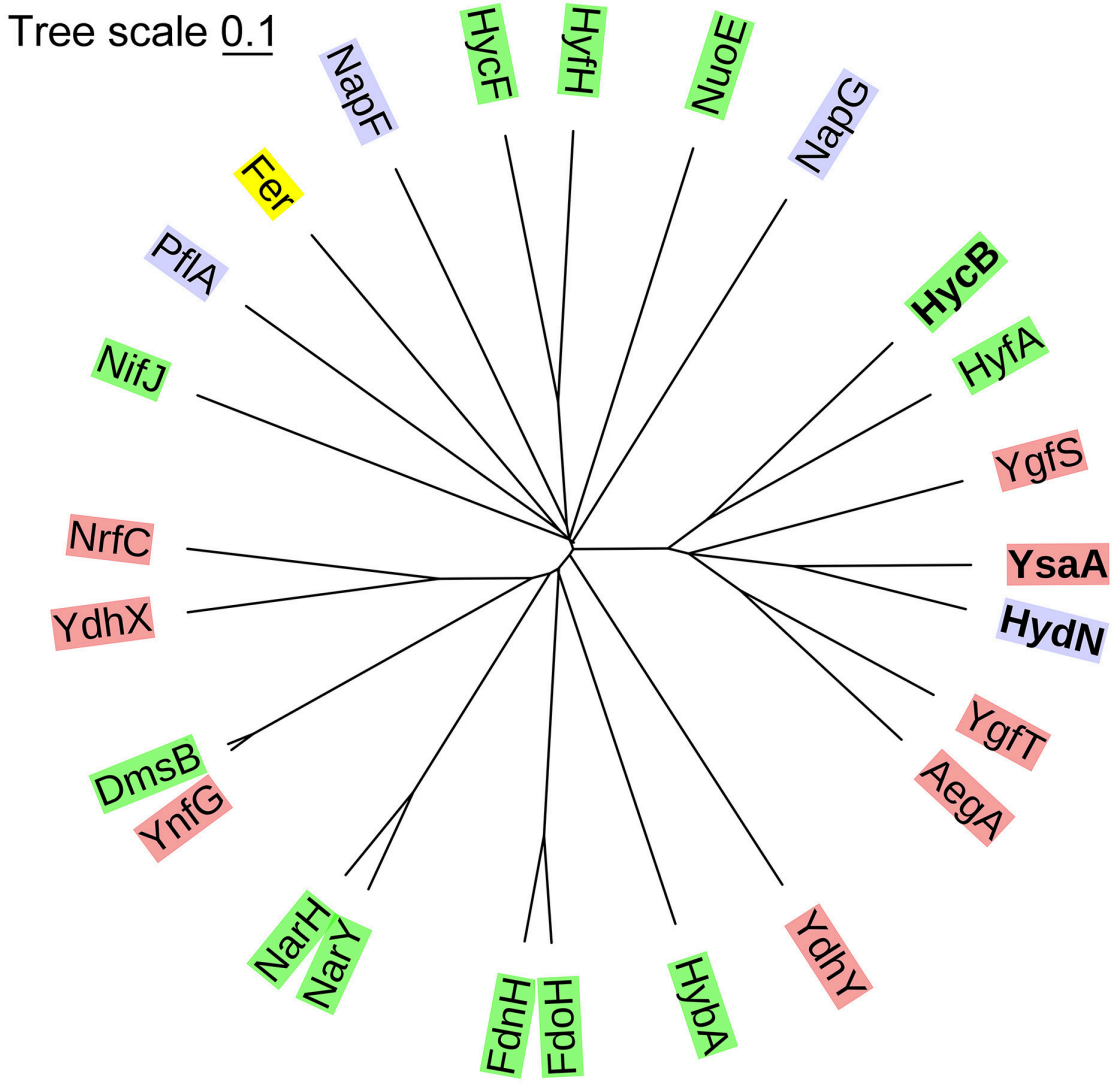
Phylogenetic tree classifying the ferredoxin-like proteins of *E. coli*. The proteins were classified based on a Clustal Omega alignment. The evolutionary distances were computed with a mBed algorithm (Sievers and Higgins, 2013) and visualized with iTOL (Letunic and Bork, 2016). The green background shows known β-subunits of respiratory complexes, in red are proteins that have no known function, blue are the proteins that are known to be required for the activity of a certain protein, but not part of the catalytically active complex and yellow is the electron carrier protein ferredoxin.

A phylogenetic tree shows that YsaA and HydN are most closely related among all known ferredoxin-like proteins in *E. coli* (Figure 1). Together with the two proteins HycB and HyfA, the small subunits of the FHL and FHL-2 complexes, respectively, and the AegA, YgfS, and YgfT proteins of unknown function, they form a group of paralogous proteins (see Figure S1 for alignment). In contrast, the HycF proteins represents the FeS-protein that provides an electron relay between the small subunit HycB of FDH-H and Hyd-3; however, HycF is more distantly related to HydN and YsaA. It is located in the tree together with proteins that are known to be required for full activity of respiratory complexes like NapF and NapG, but also with the *E. coli* ferredoxin protein Fer and the pyruvate formate-lyase activator PflA, which is required to introduce a radical into pyruvate formate-lyase (Knappe et al., 1969). The more classical β-subunits of multi-subunit respiratory enzymes like hydrogenase 2 (HybA), formate dehydrogenases N and O (subunits FdnH and FdoH, respectively), and nitrate reductases (NarH and NarY) cluster separately. Proteins of unknown function can be found clustered together both with the β-subunits and with the HydN paralogues. Due to the characteristic architecture of these proteins and the incorporation of one or more FeS clusters, it seems probable that these proteins also have a role in electron transfer within the cell.

### The Δ*ysaA* mutant has a similar phenotype to the Δ*hydN* mutant

Previous research from the Böck group had identified a *hydN* deletion strain, that resulted in reduced FDH-H activity to 38% compared to the parental, while total hydrogenase activity remained at approximately 80% (Maier et al., 1996). These findings could be reproduced in this study using both the same strain (DHD-N) used previously (Maier et al., 1996), as well as the Δ*hydN* strain (JW2683) of the Keio collection (Table 2). The FDH-H activity was reduced to 59 and 34%, while total hydrogenase activity was reduced only to 74% in the Δ*hydN* strains DHD-N and JW2683, respectively. Having an influence on the activity of an enzyme, but not completely abolishing it suggested that HydN exhibits some redundancy with another unknown protein. Therefore, the gene coding for the most similar protein YsaA was deleted by the Datsenko and Wanner method (Datsenko and Wanner, 2000) and the deletion moved into the MC4100 and DHD-N backgrounds, resulting in strains CPH010 and CPH011 (see Table 1). The strain CPH010 phenocopied the Δ*hydN* strains and had reduced FDH-H activity to 57% of the parental while total hydrogenase activity was comparable with the MC4100 parental (Table 2). Remarkably, the double deletion strain had a reduction in FDH-H activity to 28% demonstrating that the effect of the *hydN* and *ysaA* deletion was additive.

**Table 2:** Influence of YsaA and HydN on formate dehydrogenase H, hydrogenase, and H_2_-production activities.

**Strain (genotype)**	FDH-H (U ^*^ mg protein^−1^)	**% FDH-H activity of MC4100**	Total hydrogenase (U ^*^ mg protein^−1^)	H_2_-headspace (μmol ^*^ ml culture^−1^^*^${\text{OD}}_{{\text{600 nm}}^{-1}}$)
MC4100	2.81 ± 0.03	100	6.06 ± 0.05	9.7 ± 0.6
DHD-N (Δ*hydN*)	1.67 ± 0.24	59	5.87 ± 0.41	8.3 ± 0.2
JW2683 (Δ*hydN*)	0.95 ± 0.06	34	4.50 ± 0.70	9.4 ± 4.5
Δ*ysaA*	1.60 ± 0.06	57	5.93 ± 0.69	9.5 ± 0.8
Δ*aegA*	2.14 ± 0.40	76	4.18 ± 0.30	11.6 ± 2.0
Δ*hycB*	0.27 ± 0.09	10	0.25 ± 0.03	n.d.
Δ*hydN* Δ*ysaA*	0.80 ± 0.22	28	5.00 ± 0.55	8.8 ± 0.5
Δ*hydN* Δ*aegA*	0.76 ± 0.34	27	2.31 ± 0.89	10.1 ± 2.8
Δ*hydN* Δ*hycB*	0.06 ± 0.03	2	0.21 ± 0.02	n.d.
Δ*ysaA* Δ*aegA*	2.25 ± 0.15	80	4.34 ± 1.12	11.9 ± 1.9
Δ*hycB* Δ*ysaA*	0.09 ± 0.03	3	0.21 ± 0.10	n.d.
Δ*hycB* Δ*aegA*	0.11 ± 0.01	4	0.21 ± 0.05	n.d.
Δ*hycB* Δ*ysaA* Δ*aegA*	0.12 ± 0.05	4	0.19 ± 0.01	n.d.
Δ*hycB* Δ*ysaA* Δ*hydN*	0.03 ± 0.02	1	0.28 ± 0.01	n.d.
JW2683 + phydN	1.05 ± 0.18	37	2.79 ± 0.51	10.2 ± 1.9
JW2683 + pysaA	0.85 ± 0.15	30	3.52 ± 0.35	10.4 ± 1.6
DHD-N + phydN	0.97 ± 0.08	35	4.40 ± 0.39	10.1 ± 0.1
DHD-N + pysaA	0.86 ± 0.11	31	3.76 ± 0.23	8.4 ± 0.9
Δ*ysaA* + phydN	2.47 ± 0.85	88	5.71 ± 1.57	11.4 ± 0.5
Δ*ysaA* + pysaA	2.55 ± 0.60	91	5.06 ± 0.63	9.7 ± 0.4
Δ*hydN* Δ*ysaA* + phydN	1.09 ± 0.33	39	3.79 ± 0.86	10.9 ± 0.6
Δ*hydN* Δ*ysaA* + pysaA	0.77 ± 0.15	27	3.70 ± 0.14	8.3 ± 0.2

*The strains indicated were grown in TGYEP, pH 6.5 and assayed as described in the Materials and Methods section. The values are given for three independent biological replicates with standard deviations. n.d., not determined*.

Only 10% of FDH-H activity measured anaerobically as formate-dependent BV reduction remained detectable when *hycB* was deleted (Table 2). The formate-dependent BV reduction was not detectable in an *fdhF* deletion strain and therefore highly specific for FDH-H [data not shown and (Pecher et al., 1983)]. This is in agreement with a previous finding that the HycB, HycE, HycF, and HycG are equally required for full FDH-H activity (Sauter et al., 1992). In contrast to the *hydN* and *ysaA* deletion strains, the *hycB* deletion disrupted electron flow from FDH-H to hydrogenase 3 and the strain retained only residual hydrogenase activity due to hydrogenases 1 and 2 activities, and thus had a total hydrogenase activity that was reduced by more than 90% (Pinske et al., 2011b). The strain consequently failed to produce H_2_. The effect of the *hycB* deletion on FDH-H activity was even more dramatic when the *hycB* and *ysaA* deletions were combined, which resulted in a residual 3% FDH-H activity (Table 2). The same could be observed when the *hycB* and *hydN* deletions were combined in strain CPH018, which showed 2% remaining FDH-H activity and total hydrogenase activity as low as in the Δ*hycB* strain JW2694. Most strikingly, the combination of all three deletions in strain CPH019 resulted still in a detectable FDH-H activity of about 1% compared to the parental.

Electron transfer from formate to BV is thought to occur via the FeS cluster in the FDH-H polypeptide, which is located within 15 Å from the protein's surface (Boyington et al., 1997). The isolated enzyme without the small subunit was also shown to be electrochemically active in both directions on an electrode (Bassegoda et al., 2014) and in the BV assay (Axley et al., 1990). The findings in the present study, however, strongly suggest that in extracts FDH-H activity requires the presence of the ferredoxin-like proteins and in the absence of these only 1% of the FDH-H activity can be assayed directly from the enzyme.

FDH-H delivers the electrons from formate oxidation that are required for H_2_ production by the FHL complex. Alternative electron donors other than formate are not known for the FHL complex, but synthetic protein-fusion experiments with *Thermotoga maritima* ferredoxin have shown that HycB can receive electrons from other sources like pyruvate:ferredoxin oxidoreductase to some extent (Lamont et al., 2017). When cultures are grown on glucose, the H_2_ from the FHL complex accumulates in a closed growth vessel and can partially be re-oxidized by hydrogenase 1 and 2 in *E. coli*. However, in our experience the amount of accumulated H_2_ also roughly corresponds to the activity of the FHL complex and therefore is a good indicator of FHL activity (Lindenstrauß et al., 2017). By sampling the gas headspace it became clear that the *hydN* deletion reduced the FHL activity to 86% of the wild-type level, while the *ysaA hydN* double deletion retained 91% H_2_ production and the *ysaA* deletion alone had no influence on the amount of H_2_ produced (Table 2). Thus, the reduced FDH-H activity as measured by BV reduction in the *hydN* and *ysaA* deletion strains does not correlate with FHL activity as quantified by H_2_ production, perhaps suggesting that this reduced activity might be derived from FDH-H associated with another complex or complexes.

To test whether the effect of deleting the *ysaA* or *hydN* on FDH-H activity is specific, a further candidate AegA from the HydN-YsaA family of proteins was targeted and its corresponding gene *aegA* was deleted. The *aegA* gene was deleted alone and in combination with *hydN, ysaA* or *hycB*, but the effect on FDH-H activity was not greater than in the respective strains without *aegA* deletion (Table 2). Similarly, the *aegA* deletion had no influence on H_2_ production and total hydrogenase activity. This indicates that FDH-H cannot transfer electrons from formate via AegA to the redox dye BV.

Previous experiments also showed that the complementation of the *hydN* deletion did not restore full FDH-H activity (Maier et al., 1996), which could be confirmed here regardless of whether strain DHD-N or strain JW2683 was used. The Δ*hydN* Δ*ysaA* double deletion strain showed the same behavior like the two Δ*hydN* mutants after complementation with phydN. Somewhat surprisingly, restoration of almost parental FDH-H activity could be achieved when the Δ*ysaA* deletion strain was transformed with phydN (Table 2), which further substantiates at least partial redundancy of function between these proteins. The Δ*ysaA* mutant could also be complemented with pysaA to parental levels of FDH-H activity. Notably, however, introduction of the pysaA plasmid into the Δ*hydN* strain caused a further reduction of FDH-H activity. This shows that although the effect of the *hydN* and *ysaA* deletions on FDH-H activity is additive, the functions of the gene products are not identical. The use of two independent Δ*hydN* strains indicates that the lack of complementation is not strain-specific and therefore is unlikely due to additional mutations. The *hycB* deletion cannot be complemented for H_2_ production by plasmid-encoded HydN (Maier, 1997).

Based on the observation that *ysaA* and *hydN* had little influence on total hydrogenase activity and H_2_-production, the addition *in trans* of these genes was not expected to cause significant differences in these activities. The complementation with either phydN or pysaA caused a slight reduction in total hydrogenase activity in all strains tested. Another interesting effect of the complementation was visible with the GC-headspace H_2_-quantification. While the addition of pysaA had no influence on the amount of H_2_ produced by the cells in comparison to the respective plasmid-free strains, the addition of phydN increased the H_2_ amount slightly in all strains.

### Interaction network of YsaA and HydN

**Figure 2:**
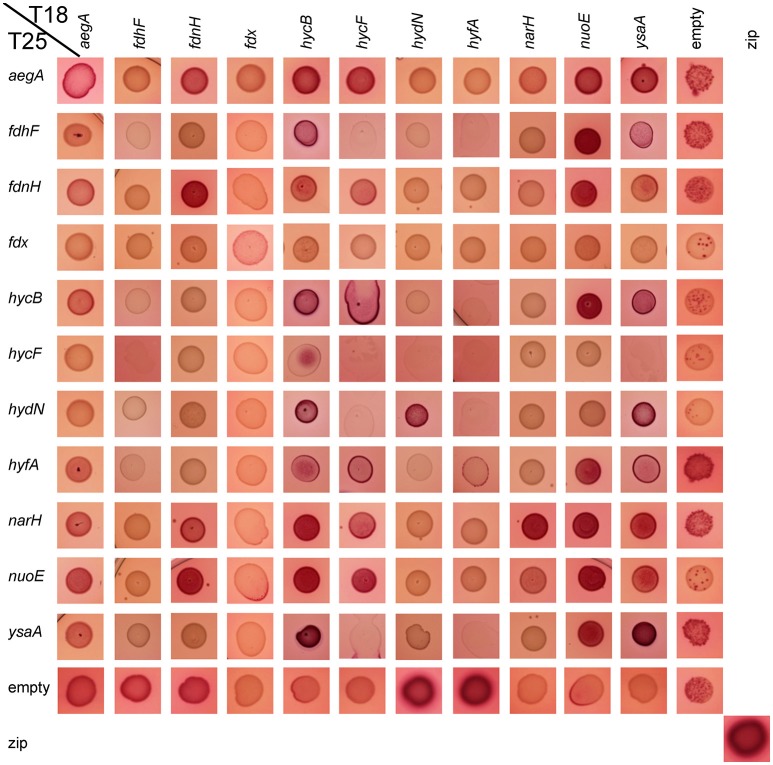
Qualitative aerobic assessment of bacterial two hybrid interactions. The respective combinations of pT18 and pT25 plasmids in strain BTH101 as given on the edges were spotted with a volume of 5 μl from liquid culture on McConkey-Agar containing 0.5% (w/v) maltose, ampicillin, and chloramphenicol and grown over night at 30°C. The red color indicates an interaction, while pale yellowish colors show a lack of interaction.

*In vivo* protein interactions can be monitored by the bacterial two-hybrid system (Karimova et al., 1998). Generally, a T18 and a T25 domain of the adenylate cyclase is fused to the target proteins *C*-terminally or *N*-terminally, respectively, and any interaction can be quantified by measurement of β-galactosidase activity. Growth of the cells under the conditions where proteins under investigation are also normally synthesized ensures that a protein interaction that requires a further unknown interaction partner can be identified. Therefore, the reporter strain was grown under anoxic conditions with glucose, where proteins of the mixed-acid fermentation are synthesized. These conditions show that empty pT25 and pUT18 plasmids yielded 217 ± 11 Miller units (MU) of activity while the zip positive control resulted in 3,619 ± 548 MU. An activity of more than 600 MU generally reflects a real interaction (Karimova et al., 1998). As additional control the empty pT25 and pT18 plasmids were co-transformed with each of the pT18 or pT25 protein fusions constructed here, respectively. They showed that the empty pT18 plasmid is not able to activate β-galactosidase in combination with any pT25 fusions. However, the empty pT25 plasmid gave a signal of more than 800 MU for the T18-HydN and T18-HyfA constructs while the other constructs showed interactions with less than the 600 MU threshold established here. Semi-aerobic interactions were additionally assessed qualitatively by spotting the colonies on McConkey-Maltose plates and showed that not all interactions identified anaerobically are detectable aerobically (Figure 2).

**Figure 3:**
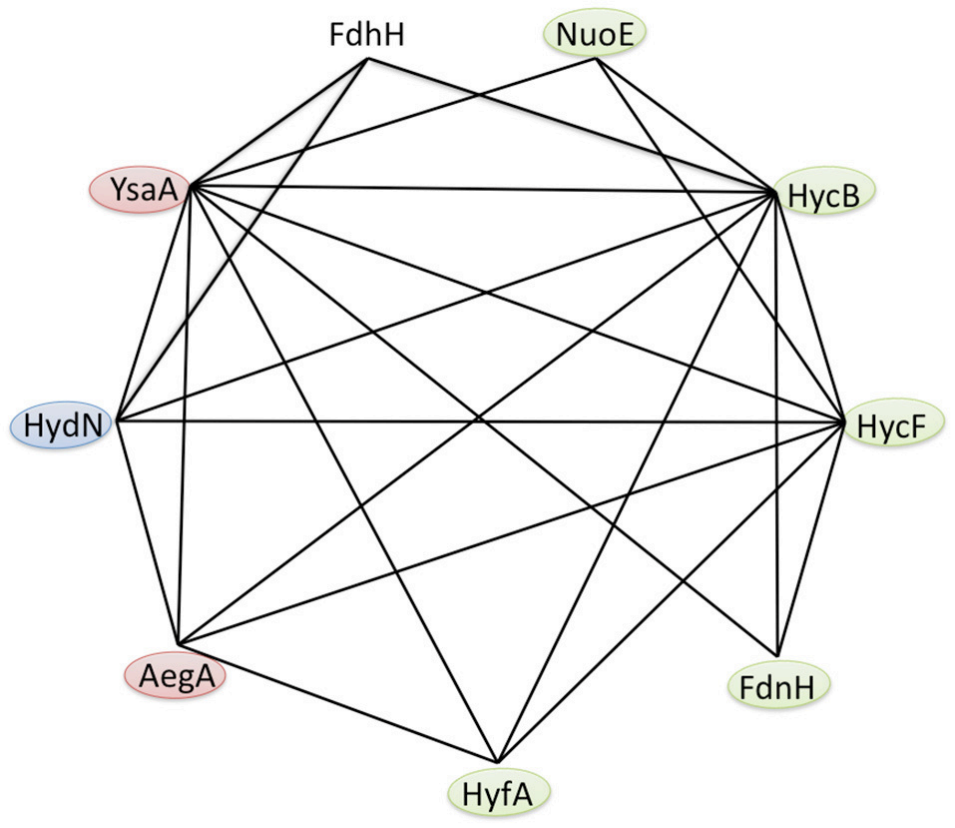
Protein interaction network of the *E. coli* ferredoxin-like family. Based on the results of the bacterial two hybrid interaction studies in Table 3, the anaerobic interactions of FDH-H and the ferredoxin-like proteins AegA, FdnH, HycB, HycF, HydN, HyfA, NuoE and YsaA are shown. Self-interactions are not visualized and the colors indicate known β-subunits (green), proteins that are required for full activity of a respiratory protein (blue) and those proteins with unknown function (red).

Initially, the interaction of FDH-H and its predicted small subunit HycB was determined. Because the *N*-terminus of this 80 kDa FDH-H protein (encoded by *fdhF*) is located at a distance of 20 Å from the FeS cluster (Boyington et al., 1997) it is therefore likely to be able to interact with its cognate small subunit. Therefore, a T25-FdhF fusion, in which the entire FDH-H protein was fused to the *C*-terminus of the T25 fragment, was constructed. The interaction with the *C*-terminal fusion of HycB (fused to T18) showed an activity of 800 MU, which was also within the same order of magnitude as the interaction with T18-HydN (728 MU) and T18-YsaA (694 MU) with T25-FdhF (Table 3). No other positive interactions of the FdhF protein were detectable here (Figure 3). The T18-HydN fusion, however, showed also an interaction with the empty T25 plasmid in this order of magnitude (892 MU) and the interaction will need to be evaluated carefully by additional methods. Although, the *C*-terminal fusion between FDH-H and HycB was previously shown to be successful in transferring electrons into the FHL complex (McDowall et al., 2015), the T18-FdhF showed neither an interaction with HycB nor with the other tested protein fusions. This could indicate that HydN and YsaA only transiently interact with FdhF before it assembles into the FHL complex, as we could show recently for the HycH protein interaction with the hydrogenase 3 of the FHL complex on the pathway of assembly into the complex (Lindenstrauß et al., 2017). But instead of a temporal order of interactions, these results could also be interpreted to indicate that FDH-H interacts with other protein complexes.

**Table 3:** Beta-galactosidase activities of bacterial two hybrid interactions.

	**T18-AegA**	**T18-FdhF**	**T18-FdnH**	**T18-Fdx**	**T18-HycB**	**T18-HycF**	**T18-HydN**	**T18-HyfA**	**T18-NarH**	**T18-NuoE**	**T18-YsaA**	**T18**
T25-AegA	982 ± 88	164 ± 19	392 ± 44	125 ± 10	1, 877 ± 164	1, 157 ± 202	2, 247 ± 336	2, 956 ± 350	164 ± 17	413 ± 27	5, 867 ± 431	62 ± 11
T25-FdhF	511 ± 153	451 ± 11	446 ± 51	132 ± 4	800 ± 37	305 ± 24	728 ± 62	338 ± 25	311 ± 149	321 ± 50	694 ± 37	40 ± 3
T25-FdnH	230 ± 39	182 ± 16	1, 282 ± 116	139 ± 19	1, 139 ± 48	1, 116 ± 45	211 ± 55	223 ± 16	278 ± 18	518 ± 37	905 ± 59	39 ± 16
T25-Fdx	107 ± 18	188 ± 27	421 ± 174	134 ± 13	500 ± 182	241 ± 166	197 ± 13	225 ± 18	267 ± 17	333 ± 112	194 ± 24	57 ± 13
T25-HycB	6, 482 ± 771	421 ± 12	1, 111 ± 37	127 ± 9	2, 413 ± 176	3, 973 ± 420	4, 544 ± 115	265 ± 19	429 ± 252	1, 334 ± 50	4, 215 ± 303	30 ± 15
T25-HycF	1, 555 ± 175	300 ± 125	1, 109 ± 68	141 ± 11	2, 174 ± 155	2, 505 ± 375	1, 683 ± 191	341 ± 141	448 ± 127	311 ± 45	2, 188 ± 185	41 ± 14
T25-HydN	4, 581 ± 788	401 ± 23	450 ± 52	141 ± 10	3, 131 ± 146	2, 120 ± 286	3, 172 ± 116	317 ± 20	282 ± 22	363 ± 182	3, 072 ± 116	17 ± 10
T25-HyfA	1, 773 ± 277	272 ± 8	423 ± 21	152 ± 25	1, 066 ± 54	2, 567 ± 219	449 ± 44	296 ± 12	491 ± 194	537 ± 59	2, 660 ± 96	48 ± 9
T25-NarH	257 ± 24	195 ± 33	1, 255 ± 77	140 ± 7	1, 283 ± 148	1, 211 ± 89	186 ± 15	206 ± 21	1, 084 ± 137	1, 481 ± 166	927 ± 220	29 ± 6
T25-NuoE	417 ± 45	183 ± 16	512 ± 38	158 ± 19	1, 546 ± 97	1, 273 ± 153	194 ± 22	243 ± 137	472 ± 136	1, 450 ± 84	618 ± 66	65 ± 6
T25-YsaA	6, 150 ± 1, 172	410 ± 18	446 ± 61	150 ± 14	3, 896 ± 194	2, 370 ± 180	4, 608 ± 519	944 ± 30	297 ± 26	308 ± 22	4, 237 ± 268	63 ± 8
T25	400 ± 62	590 ± 70	330 ± 26	244 ± 8	161 ± 12	341 ± 45	892 ± 39	821 ± 60	336 ± 17	148 ± 11	146 ± 18	217 ± 11

a *Shown are the mean Miller Units (MU) from 3 independent biological samples measured in duplicates to triplicates with their respective standard deviations. Cells were grown anaerobically in TGYEP, pH 6.5 until stationary phase. The zip plasmids and empty plasmids served as positive and negative controls, respectively (Karimova et al., 1998). The positive control yielded an activity of 3,619 ± 548 MU and empty pUT18 and pT25 vectors as negative control had an activity of 217 ± 11 MU Strong interactions (> 1,000 MU) are highlighted by an orange color, weak interactions (600–1,000 MU) are shown with a yellow background*.

The strong interaction between the *N*-terminal fusions of HydN, YsaA and the HycB proteins and their corresponding *C*-terminal fusions favors the latter hypothesis (Table 3). These range between 2,413 MU for the HycB self-interaction and 4,608 MU for the T25-YsaA × T18-HydN interaction. Interestingly, the AegA protein participates in this interaction network by showing strong interactions with itself, HycB, HydN, and YsaA. The close proximity of the second ferredoxin-like protein in the FHL complex HycF to HycB is probably the reason this protein interacts with the same partners as HycB, with the exception of FDH-H. It is striking that no interaction between the HycB homolog, HyfA, of the FHL-2 complex and the FDH-H can be detected here. Although a missing bacterial two hybrid interaction does not necessarily reflect the absence of a true interaction, the similarity of the HycB and HyfA proteins and the detectable interaction of the former, is a strong indication and these initial findings do not support the proposed interaction (Bagramyan and Trchounian, 2003) between FDH-H with the HyfA subunit of the FHL-2 complex. It should be noted, however, that AegA and YsaA interact in only one orientation with HycB and HycF of the FHL complex. Thus, further fusion constructs need to be tested.

It is noteworthy that YsaA also showed an interaction with FdnH and NarH, the two β-subunits of nitrate-dependent formate dehydrogenase (FDH-N) and nitrate reductase (NAR), respectively. The genes encoding these enzymes are transcribed at only a low level (Walker and DeMoss, 1991; Li and Stewart, 1992), therefore these subunits of FDH-N and NAR complexes are likely to be present in the assay. The HydN and AegA proteins do not interact with these nitrate-dependent proteins, supporting the specificity for the YsaA interaction. The FdnH and NarH proteins furthermore show a self-interaction as well as a cross-interaction, which can be explained for FdnH because the enzyme complex FdnGHI exists as a trimer of trimers (Jormakka et al., 2003) and although primarily coupled by a Q-cycle the special co-localisation of FDH-N and NAR activities was shown to be highly organized and proximal to each other in the membrane (Alberge et al., 2015).

The previously identified interaction between YsaA with NuoE, a subunit of Complex I, using Tap-tag technology could also be verified here (Arifuzzaman et al., 2006). Further interactions of NuoE with itself and NarH, as well as of NuoE with the FHL subunits HycB and HycF were also detected here. However, the Fdx protein did not show any interaction with the protein fusions tested here, showing that its interaction with the proteins of the FeS-insertion machinery of the cell is highly specific (Tokumoto et al., 2002). This further indicates that it is not the general fold of the protein that is important for the interaction, but particular residues on the surface of the respective protein. Moreover, there are significant differences in length in a loop region between the third and the fourth Cys-rich (Cx_2_Cx_2−8_Cx_3_C) motif and the *C*-terminal region of the HycB, HydN, and YsaA proteins, the former lies at the expected interface with the interaction partner and this loop could also play a role in discrimination of the correct target. All identified interactions are summarized in Figure 3.

### FDH-H protein pattern remains unaltered

**Figure 4:**
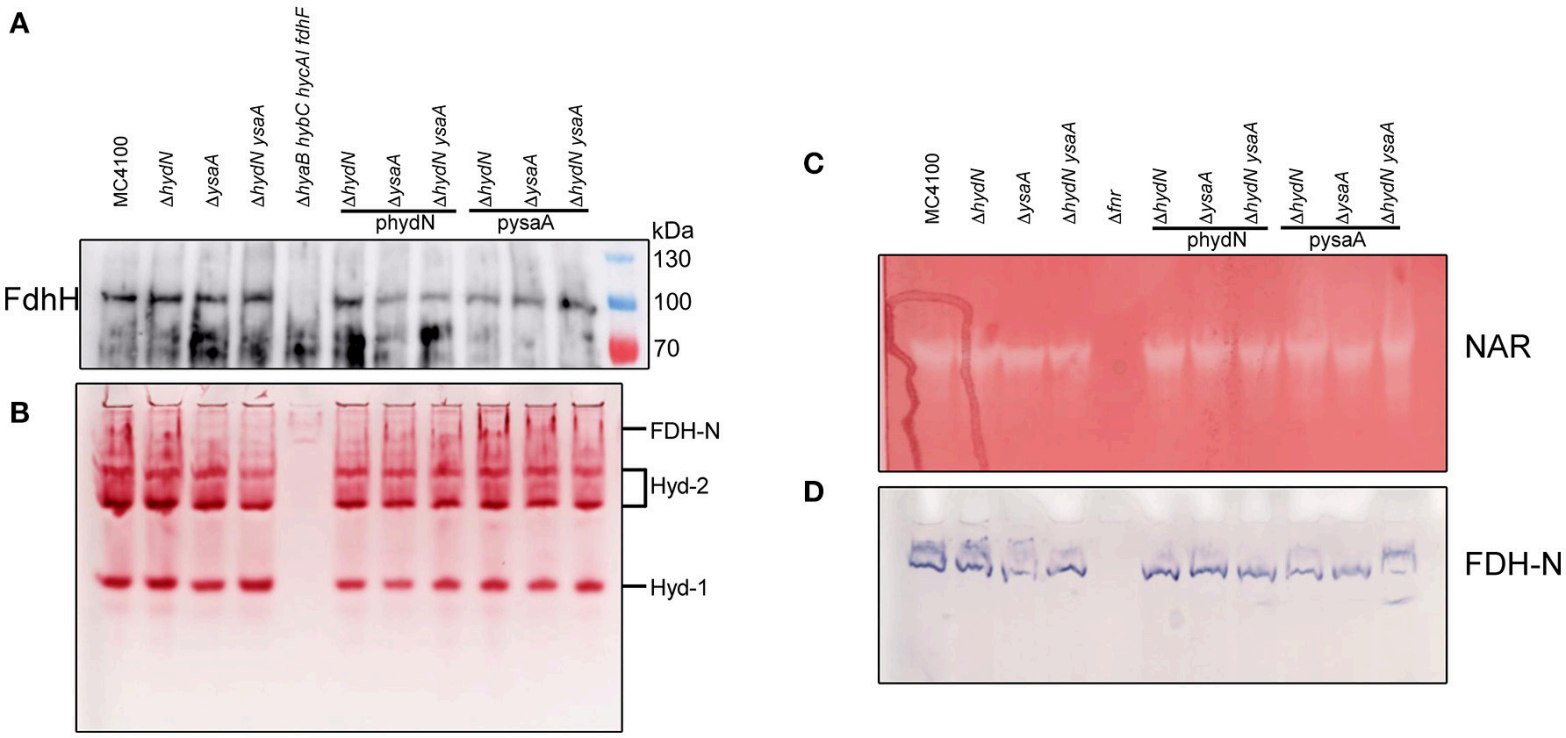
Influence of HydN and YsaA on activities and polypeptides. **(A)** Shows a western blot of anaerobically grown cells as indicated by their genotype after separation of 25 μg protein in a SDS-PAGE, transfer to nitrocellulose and challenge with antibodies against FDH-H. **(B)** Shows identical samples but non-denatured and separated in a native-PAGE and stained for hydrogenase activity with H_2_/BV and TTC. **(C**,**D)** Show a native-PAGE of cells as indicated by their genotype on top after anaerobic growth with 1% nitrate and staining for NAR (nitrate, BV, TTC, dithionite) and FDH-N (PMS, NBT, formate) activities, respectively.

Remarkably, although the activity of FDH-H varies among the Δ*hydN* and Δ*ysaA* strains, western blot analysis of the polypeptide showed that both the migration pattern and the protein amount appear similar in the *ysaA, hydN*, and *ysaA hydN* deletion mutants when compared to the protein in the MC4100 parental strain; the same is the case for strains carrying the plasmids pysaA or phydN (Figure 4A). Therefore, the transcription or translation of the *fdhF* gene is not altered in the mutants.

A native PAGE showing the activities of hydrogenases 1 and 2 (Figure 4B) reveals that the migration pattern and in-gel enzyme activities are similar regardless of the presence or absence of either HydN or YsaA. These hydrogenases harbor the same [NiFe]-cofactor as HycE, the hydrogenase of the FHL complex. Thus the influence is strictly limited to FDH-H and not the hydrogenases in general.

### The *hydN* and *ysaA* deletions have no influence on other Mo-bis-PGD–dependent activities

In order to investigate whether the influence of the *hydN* and *ysaA* mutations on FDH-H is generally related to Mo-bis-PGD cofactor insertion, the cytoplasmic NAR and periplasmic FDH-N activities, which are both Mo-bis-PGD-dependent, were analyzed. Figures 4C,D show that the amount and migration patterns of the nitrate reductase and formate dehydrogenase N activities were comparable to those in the wild type strain, regardless of whether HydN and YsaA are present or not. Both activities are strictly dependent on Fnr for gene expression, hence the Δ*fnr* mutant RM102 served as a negative control.

## Conclusions

Of the three formate dehydrogenases encoded in the *E. coli* genome, FDH-H is the only cytoplasmic enzyme. An early mutant study by Mandrand-Berthelot showed that differences in the requirements for maturation of the cytoplasmic and periplasmic formate dehydrogenases exist (Mandrand-Berthelot et al., 1988). One of the isolated mutants had a mutation in *fdhE* and was deficient in the respiratory formate dehydrogenases N and O only; this mutation was without effect on transcription (Stewart et al., 1991). The mutation in another mutant was located in *fdhD* and had an effect on all three formate dehydrogenases, because FdhD, as was shown recently (Thomé et al., 2012; Arnoux et al., 2015), is involved in donating a sulfur ligand to the Mo-bis-PGD. It is conceivable that HydN and YsaA could contribute specifically to the cofactor activation of the FDH-H protein, since NAR and FDH-N activities are not impaired in the mutant; however, it would be expected that a more pronounced phenotype of the individual mutants would be observed, making this explanation unlikely.

Based on the observation of reduced, but not completely abolished FDH-H activity in the *ysaA, hydN, hycB* and the double and triple deletion mutants, it appears that full redox dye-reducing activity of FDH-H can only be measured when all three proteins are present simultaneously. Therefore, either all three proteins are involved as components of the FHL complex, although only HycB is essential (Sauter et al., 1992; Table 2) or HydN and YsaA could represent alternative β-subunits of the FDH-H enzyme, linking formate oxidation to other protein complexes. Neither HydN nor YsaA could be identified as components of the purified FHL complex (McDowall et al., 2014). If HydN and YsaA were transient components of FHL, then it would be anticipated that additional copies of *hydN in trans* should compete for FDH-H protein with HycB and reduce the H_2_-production by the FHL complex. However, H_2_ production by the FHL complex remained essentially unaffected by all these mutations, which renders this hypothesis improbable. The data currently suggest, therefore, that HydN/YsaA provide alternative electron transfer routes for FDH-H to non-hydrogen producing electron acceptor complexes.

The phylogenetic analysis shows that AegA and YgfST are closely related to YsaA and HydN. Although AegA interacts with the ferredoxin-like proteins, the lack of interaction with FDH-H reflects an absence of influence on FDH-H activity. The AegA protein is a fusion of two domains; an *N*-terminal ferredoxin-like domain and a *C*-terminal glutamate synthase domain (Cavicchioli et al., 1996). Identifying its role within the network of ferredoxin-like proteins will require further experiments.

Generally, these interaction data have to be carefully evaluated because some proteins require interaction partners and if the physiological partner is not present, they seem to interact non-specifically with a number of other proteins. Therefore, future protein-protein interaction experiments will have to be performed *in vitro*. So far, HydN has proved recalcitrant to over-production and alternative purification strategies will have to be established to allow full biochemical characterization of this protein family.

## Author contributions

CP planned and performed the experiments. CP has drafted and written the manuscript.

### Conflict of interest statement

The author declares that the research was conducted in the absence of any commercial or financial relationships that could be construed as a potential conflict of interest.

## Abbreviations

FHL: formate hydrogenlyase
Mo-bis-PGD: molybdopterin guanine dinucleotide
BV: benzyl viologen
FDH-H: formate dehydrogenase H
MU: Miller Units.
