# Supplementary material for: The Ferredoxin-Like Proteins HydN and YsaA Enhance Redox Dye-Linked Activity of the Formate Dehydrogenase H Component of the Formate Hydrogenlyase Complex
Source: Front Microbiol. 2018 Jun 11;9:1238. doi: 10.3389/fmicb.2018.01238 (PMC6004506; doi:10.3389/fmicb.2018.01238)
Supplement: Supplementary file 1 [file Table_1.DOCX]

**The Ferredoxin-like Proteins HydN and YsaA Enhance Redox Dye-linked Activity of the Formate Dehydrogenase H Component of the Formate Hydrogenlyase Complex**

Constanze Pinske*

Institute for Biology/Microbiology, Martin-Luther University Halle-Wittenberg, Kurt-Mothes-Str. 3, 06120 Halle/Germany

**Supplemental Information**

**Table S1: Oligonucleotides used for cloning of bacterial two hybrid constructs.**

|  | target vector | |
| --- | --- | --- |
| gene | pUT18^a^ | pT25^a^ |
| *aegA* | T18-aegAFW_HindIII  gcg AAGCTT g atgAATCGTTTTATTATGGC  T18-aegARW_EcoRI  gcg GAATTC ga GTGAGATTTGACTGATTTTAC | T18C/T25-aegAFW_BamHI  gcg GGATCC c AATCGTTTTATTATGGCCAAC  T18C/T25-aegARW_KpnI  gcg GGTACC tcaGTGAGATTTGACTGATTTTAC |
| *fdhF* | FdhF_pUT18FW_HindIII  gcg AAGCTTg atgAAAAAAGTCGTCACGGT  HycB_pUT18RW_EcoRI  gcg GAATTC ga CGCCAGTGCCGCTTCGCGCA | FdhF_pT25FW_PstI  gcg CTGCAGgg AAAAAAGTCGTCACGGTTTG  FdhF_pT25RW_BamHI  gcg GGATCC ttaCGCCAGTGCCGCTTCGC |
| *fdnH* | T18-fdnHFW_HindIII  gcg AAGCTT g atgGCTATGGAAACGCAGG  T18-fdnHRW_EcoRI  gcg GAATTC gaCTCATGATGATCCTCCTCGTC | T18C/T25-fdnHFW_BamHI  gcg GGATCC cGCTATGGAAACGCAGGACAT  T18C/T25-fdnHRW_KpnI  gcg GGTACC ttaCTCATGATGATCCTCC |
| *fdx* | T18-fdxFW_HindIII  gcg AAGCTT g atgCCAAAGATTGTTATTTTG  T18-fdxRW_EcoRI  gcg GAATTC gaATGCTCACGCGCATGGTTG | T18C/T25-fdxFW_BamHI  gcg GGATCC c CCAAAGATTGTTATTTTGCC  T18C/T25-fdxRW_KpnI  gcg GGTACC ttaATGCTCACGCGCATGGT |
| *hycB* | HycB_pUT18FW_HindIII  gcg AAGCTTg atgAATCGTTTTGTAATTGCTG  HycB_pUT18RW_EcoRI  gcg GAATTC ga TTTAGCCTCTCCACTTTGAG | HycB_pT25FW_PstI  gcg CTGCAGgg AATCGTTTTGTAATTGCTGAC  HycB_pT25RW_BamHI  gcg GGATCC tcaTTTAGCCTCTCCACTTT |
| *hycF* | HycF_pUT18_PstI  gcg CTGCAGg atgTTTACCTTTATCAAAAAAG  HycF_pUT18_EcoRI  gcg gaattc gaGATGGCCTCTTTCATATGGC | HycF_pT25FW_PstI  gcg CTGCAGgg TTTACCTTTATCAAAAAAGT  HycF_pT25RW_BamHI  gcg GGATCC tcaGATGGCCTCTTTCATATG |
| *hydN* | HydN_pUT18FW_HindIII  gcg AAGCTTg atgAACCGTTTCATCATTGC  HydN_pUT18RW_EcoRI  gcg GAATTCga GAACATCAGCGCCGTACGGC | HydN_pT25FW_PstI  gcg CTGCAGgg AACCGTTTCATCATTGCTGAC  HydN_pUT18CRW_BamHI  gcg GGATCC ttaGAACATCAGCGCCGTAC |
| *hyfA* | HyfA_T18FW_KpnI  gcg GGTACC atgAACCGCTTTGTGGTGGC  HyfA_T18RW_EcoRI  gcg GAATTC ga GCGTTGCTCCTGAGTGAGGG | HyfA_PT25FW_KpnI  gcg GGTACC t AACCGCTTTGTGGTGGCCGA  HyfA_pT25RW_KpnI  gcg GGTACC ttaGCGTTGCTCCTGAGTGAG |
| *narH* | T18-narHFW_HindIII  gcg AAGCTT g atgAAAATTCGTTCACAAG  T18-narHRW_EcoRI  gcg GAATTC ga TGGATGCGGCTCCGTTTTGC | T18C/T25-narHFW_KpnI  gcg GGTACC t AAAATTCGTTCACAAGTCGG  T18C/T25-narHRW_KpnI  gcg GGTACC tcaTGGATGCGGCTCCGTTTTG |
| *nuoE* | T18-nuoEFW_HindIII  gcg AAGCTT g atgCACGAGAATCAACAACC  T18-nuoERW_EcoRI  gcg GAATTC ga TTTATACCGCTCCAGCAGTTC | T18C/T25-nuoEFW_BamHI  gcg GGATCC c CACGAGAATCAACAACCACA  T18C/T25-nuoERW_KpnI  gcg GGTACC tta tcaTTTATACCGCTCCAGCAG |
| *ysaA* | T18-ysaAFW_HindIII  gcg AAGCTT g atgAACCGGTTTATTATTGCG  T18-ysaARW_EcoRI  gcg GAATTC ga AACAGGCTGCTGCCGTAGCC | T18C/T25-ysaAFW_BamHI  gcg GGATCC c AACCGGTTTATTATTGCGGAT  T18C/T25-ysaARW_KpnI  gcg GGTACC tta tcaAACAGGCTGCTGCCGTAGC |

^a^The box contains the name of the forward oligonucleotide followed by its sequence in 5’ to 3’ direction and then the same for the reverse oligonucleotide.

**Table S2:** Plasmids of the bacterial two hybrid system constructed here.

| Plasmid | Genotype | Source |
| --- | --- | --- |
| pT18-AegA | pT18, AegA-T18 fusion protein, *aegA* cloned HindIII/EcoRI, Amp^R^ | This work |
| pT18-FdhF | pT18, FdhF-T18 fusion protein, *fdhF* cloned HindIII/EcoRI, Amp^R^ | This work |
| pT18-FdnH | pT18, FdnH-T18 fusion protein, *fdnH* cloned HindIII/EcoRI, Amp^R^ | This work |
| pT18-Fdx | pT18, Fdx-T18 fusion protein, *fdx* cloned HindIII/EcoRI, Amp^R^ | This work |
| pT18-HycB | pT18, HycB-T18 fusion protein, *hycB* cloned HindIII/EcoRI, Amp^R^ | This work |
| pT18-HycF | pT18, HycF-T18 fusion protein, *hycF* cloned PstI/EcoRI, Amp^R^ | This work |
| pT18-HydN | pT18, HydN-T18 fusion protein, *hydN* cloned HindIII/EcoRI, Amp^R^ | This work |
| pT18-HyfA | pT18, HyfA-T18 fusion protein, *hyfA* cloned KpnI/EcoRI, Amp^R^ | This work |
| pT18-NarH | pT18, NarH-T18 fusion protein, *narH* cloned HindIII/EcoRI, Amp^R^ | This work |
| pT18-NuoE | pT18, NuoE-T18 fusion protein, *nuoE* cloned HindIII/EcoRI, Amp^R^ | This work |
| pT18-YsaA | pT18, YsaA-T18 fusion protein, *ysaA* cloned HindIII/EcoRI, Amp^R^ | This work |
| pT25-AegA | pT25, T25-AegA fusion protein, *aegA* cloned BamHI/KpnI, Cm^R^ | This work |
| pT25-FdhF | pT25, T25-FdhF fusion protein, *fdhF* cloned PstI/BamHI, Cm^R^ | This work |
| pT25-FdnH | pT25, T25-FdnH fusion protein, *fdnH* cloned BamHI/KpnI, Cm^R^ | This work |
| pT25-Fdx | pT25, T25-Fdx fusion protein, *fdx* cloned BamHI/KpnI, Cm^R^ | This work |
| pT25-HycB | pT25, T25-HycB fusion protein, *hycB* cloned PstI/BamHI, Cm^R^ | This work |
| pT25-HycF | pT25, T25-HycF fusion protein, *hycF* cloned PstI/BamHI, Cm^R^ | This work |
| pT25-HydN | pT25, T25-HydN fusion protein, *hydN* cloned PstI/BamHI, Cm^R^ | This work |
| pT25-HyfA | pT25, T25-HyfA fusion protein, *hyfA* cloned KpnI/KpnI, Cm^R^ | This work |
| pT25-NarH | pT25, T25-NarH fusion protein, *narH* cloned KpnI/KpnI, Cm^R^ | This work |
| pT25-NuoE | pT25, T25-NuoE fusion protein, *nuoE* cloned BamHI/KpnI, Cm^R^ | This work |
| pT25-YsaA | pT25, T25-YsaA fusion protein, *ysaA* cloned BamHI/KpnI, Cm^R^ | This work |

**Figure S1: Alignment of the HydN group of ferredoxin-like proteins.** The proteins with the given ID’s were aligned with the ClustalO algorithm and visualized with Jalview. Cysteines were highlighted in yellow. Regions where only one protein without cysteines was shown, were omitted from the alignment. The bars under the alignment indicate Conservation, Quality, Consensus and Occupancy of the alignment.
