# Supplementary figures and images for: The Ferredoxin-Like Proteins HydN and YsaA Enhance Redox Dye-Linked Activity of the Formate Dehydrogenase H Component of the Formate Hydrogenlyase Complex
Source: Front Microbiol. 2018 Jun 11;9:1238. doi: 10.3389/fmicb.2018.01238 (PMC6004506; doi:10.3389/fmicb.2018.01238)

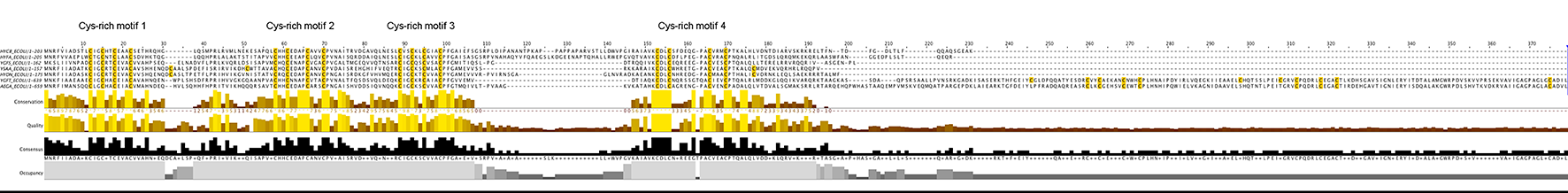

Supplement: Supplementary file 2 [file Image_1.TIF]
